# The phenotype of MEGF8-related Carpenter syndrome (CRPT2) is refined through the identification of eight new patients

**DOI:** 10.1038/s41431-024-01624-9

**Published:** 2024-05-17

**Authors:** Laura M. Watts, Marta Bertoli, Tania Attie-Bitach, Natalie Roux, Antonio Rausell, Cate R. Paschal, Jessica L. Zambonin, Cynthia J. Curry, Blanche Martin, Rebecca S. Tooze, Lara Hawkes, Usha Kini, Stephen R. F. Twigg, Andrew O. M. Wilkie

**Affiliations:** 1 Oxford Centre for Genomic Medicine, Oxford University Hospitals NHS Foundation Trust, Oxford, UK; 2 Northern Genetics Service, Newcastle upon Tyne Hospitals NHS Foundation Trust, Newcastle upon Tyne, UK; 3 Service de Médecine Génomique des Maladies Rares, Hôpital Necker-Enfants Malades, Paris, France; 4 Laboratoire de biologie médicale multisites SeqOIA, Paris, France; 5 Seattle Children’s Hospital, Seattle, WA USA; 6 Provincial Medical Genetics Program, BC Women’s Hospital and Health Centre, Vancouver, BC Canada; 7 University of California San Francisco/Fresno, Fresno, CA USA; 8 Genetic Medicine, Community Regional Medical Center, Fresno, CA USA; 9 MRC Weatherall Institute of Molecular Medicine, University of Oxford, John Radcliffe Hospital, Oxford, UK; 10 Present Address: Institut de Pathologie et de Génétique, Gosselies, Belgium

**Keywords:** Disease genetics, Genetics research

## Abstract

Carpenter syndrome (CRPTS) is a rare autosomal recessive condition caused by biallelic variants in genes that encode negative regulators of hedgehog signalling (*RAB23* [CRPT1] or, more rarely, *MEGF8* [CRPT2]), and is characterised by craniosynostosis, polysyndactyly, and other congenital abnormalities. We describe a further six families comprising eight individuals with *MEGF8*-associated CRPT2, increasing the total number of reported cases to fifteen, and refine the phenotype of CRPT2 compared to CRPT1. The core features of craniosynostosis, polysyndactyly and (in males) cryptorchidism are almost universal in both CRPT1 and CRPT2. However, laterality defects are present in nearly half of those with *MEGF8*-associated CRPT2, but are rare in *RAB23*-associated CRPT1. Craniosynostosis in CRPT2 commonly involves a single midline suture in comparison to the multi-suture craniosynostosis characteristic of CRPT1. No patient to date has carried two *MEGF8* gene alterations that are both predicted to lead to complete loss-of-function, suggesting that a variable degree of residual MEGF8 activity may be essential for viability and potentially contributing to variable phenotypic severity. These data refine the phenotypic spectrum of CRPT2 in comparison to CRPT1 and more than double the number of likely pathogenic *MEGF8* variants in this rare disorder.

## Introduction

Carpenter syndrome (CRPTS) is an autosomal recessive disorder characterised by the core features of craniosynostosis and polysyndactyly of the hands and feet, with other reported features including obesity, congenital heart disease and cryptorchidism [1]. Originally described in 1901 in a family with craniosynostosis and polysyndactyly [2], the most common form of CRPTS (CRPT1; OMIM 201000) was shown in 2007 to be caused by biallelic pathogenic variants in *RAB23* [3, 4]. *RAB23* encodes a small GTPase that is a negative regulator of hedgehog signalling and loss of RAB23 function causes excessive hedgehog signalling resulting in craniosynostosis and polydactyly. Although the precise mechanism by which RAB23 represses hedgehog signalling remains unclear, there is evidence for differential regulation by RAB23 of downstream GLI transcription factors (repression of GLI2, activation of GLI3 repressor) [3, 5–8]. Since the original description of *RAB23*-associated CRPTS in 17 affected individuals [3], a further 22 cases have been described with common additional features including facial dysmorphism, short stature with obesity, cryptorchidism, congenital heart disease and variable developmental delay [4, 9–15]. Rarely, patients heterozygous for megabase-sized deletions of chromosome 7p14.1, including *GLI3* and multiple contiguous genes, may present with a phenotype that overlaps with CRPTS [16, 17].

In 2012 a second genetic type of CRPTS (CRPT2; OMIM 614976) was delineated, caused by biallelic alterations in the gene encoding multiple epidermal-growth-factor-like-domains 8 (*MEGF8*). The initial report featured five affected individuals from four families, who harboured biallelic variants (missense, nonsense or splicing) in *MEGF8*. Analysis of the missense variants in a zebrafish embryo assay supported that these were deleterious for protein function. Although substantial clinical overlap was observed with *RAB23*-associated CRPTS, abnormal left-right patterning was more frequent (3/5 cases) [18] and mirrored observations in mice. Mice with alterations in *Megf8* have polydactyly and altered left-right patterning, similar to those found in humans [19–21], whilst morpholino knockdown of *megf8* in zebrafish also results in alterations in left-right patterning [19]. Mechanisms underlying the phenotype are unclear, although similar to RAB23, evidence supports MEGF8 as a negative regulator of hedgehog signalling [22]. In contrast to RAB23, MEGF8 appears to act by promoting degradation of the hedgehog pathway signal transduction factor Smoothened [6, 22].

Since the original description of *MEGF8*-associated CRPTS in 2012, we are only aware of one other family, comprising two affected siblings, formally reported in the literature [23]. Owing to the paucity of reported cases, evidence for CRPT2 was classified as being Moderate using the ClinGen Craniofacial Malformations gene-disease curation process [24, 25]. Here we describe a further eight affected individuals (thus more than doubling the number of documented cases of this rare pathological entity) and compare these to previously reported cases of *MEGF8*- and *RAB23*-associated CRPTS, emphasising that the core clinical features are similar between *RAB23* and *MEGF8* patients, but demonstrating that laterality defects are more common, and craniosynostosis appears less severe, in the *MEGF8*-associated form of the condition.

## Patients and methods

A single family (Family 6) was formally enrolled into a research study (Genetic Basis of Craniofacial Malformation; London Riverside NHS Research Ethics Committee ref 09/H0706/20). For the remaining families, clinicians who had either previously contacted the Clinical Genetics Group at the MRC Weatherall Institute of Molecular Medicine Oxford, regarding patients with *MEGF8* alterations, or presented unpublished data at specialist meetings, were re-contacted to ascertain whether the patient or parents would be willing to participate in the study. Following agreement, the clinician was sent a proforma to complete providing clinical details including *MEGF8* alteration, details of craniosynostosis, hand and foot abnormalities, and cardiac, gastrointestinal and developmental findings. All participants or their legal guardians consented to inclusion in this study.

*MEGF8* has two major splice forms, which differ depending upon the inclusion or exclusion of exon 12A, without altering the reading frame [18]. For comparison with the previous publication all variant details are numbered with respect to the shorter 2778 amino acid reference transcript (RefSeq accession NM_001410.3, Ensembl ENST00000334370.8); note that the MANE select transcript is the longer isoform encoding 2845 amino acids, which, however, shows low expression in most or all tissues (Genotype-tissue expression project analysis release V8 [26]).

## Results

We describe eight individuals from six unrelated families with clinical features compatible with CRPTS. Each family presented to a different clinical centre, and following diagnostic exome or genome sequencing, was found to harbour two variants in *MEGF8* consistent with a causal role. The major molecular and clinical features in these individuals are summarised in Table 1 and their clinical presentations are outlined below. A more detailed clinical description of each patient is provided in the Supplementary Case Descriptions and Supplementary Table 1. To provide a comparison with previously reported cases of *MEGF8*- and *RAB23*-associated CRPTS, a summary of all cases is presented in Supplementary Table 2.

**Table 1:** Summary of clinical and molecular features of the newly reported MEGF8 Carpenter syndrome cases.

Family	Sex	Age at examination	Variants (NM_001410.3)	Inheritance	CADD score	gnomAD frequency	Craniosynostosis	Facial dysmorphism	Brachydactyly	Syndactyly	Polydactyly	Talipes equinovarus	Laterality defect	Cardiac findings	Genital abnormalities	Developmental delay
1	M	37 years	c.4496G>A p.(Arg1499His)	Paternal	27	8.26 × 10^−6^	Y (sutures unknown)	+	+	H (1–5 BL) & Fo (1–5 BL)	Fo	−	−	−	Cryptorchidism (L)	−
			c.7766_7768del p.(Phe2589del)	Maternal	–	0										
2.i	F	25 weeks’ gestation	c.878T>C p.(Leu293Pro)	Paternal	28	1.45 × 10^−6^		+	−	H	−	+	+	Left atrial isomerism, AVSD, bilateral SVCs	−	NA
			c.2298+1G>A p.?	Maternal	34	6.86 × 10^−6^										
2.ii	M	7 days (initial); 17 months	c.878T>C p.(Leu293Pro)	Paternal	28	1.45 × 10^−6^	S	+	+	H (1–5 BL) & Fo (1–5 BL)	−	−	+	DORV, ASD,VSD, PDA	Cryptorchidism	+
			c.2298+1G>A p.?	Maternal	34	6.86 × 10^−6^										
3	M	6 weeks	c.5643+1G>A	De novo^a^	34	1.38 × 10^−6^	Me	+	+	H (1–5 BL) & Fo (1–5 BL)	Fo	+	−	PDA	Cryptorchidism	+
			c.8137G>A p.(Val2713Met)	Maternal	25.9	2.14 × 10^−6^										
4	F	2 years	c.4872delG p.(Phe1625Serfs*38)	Paternal or De novo^b^	34	1.38 × 10^−6^	Me	+		H (1–5 BL)	Fo	−	−	ASD, PDA	−	+
			c.7769_7771delCCT p.(Ser2590del)	Maternal	–	0										
5	F	20 weeks’ gestation	c.7759_7770del p.(Val2587_Ser2590del)	Paternal	–	0	All	+	Thumb brachymetacarpy	H & Fo	H & Fo	−	+	Left atrial isomerism	Uterine agenesis, streak ovaries	NA
			c.7791_7794del p.(Leu2598Valfs*24)	Maternal	33	6.88 × 10^−7^										
6.i	M	16 years	c.7126C>T (p.Arg2376Cys)	Paternal	28	2.51 × 10^−6^	Me, S	+	Spatulate thumbs with split distal phalanx	H & Fo	Fo	+	−	−	Cryptorchidism (BL)	+
			c.7068+5G>A	Maternal	22.7	3.1 × 10^−6^										
6.ii	M	10 years	c.7126C>T (p.Arg2376Cys)	Paternal	28	2.51 × 10^−6^	–	+	−	H(2–5) & Fo	−	−	−	VSD	Cryptorchidism (BL)	+
			c.7068+5G>A	Maternal	22.7	3.1 × 10^−6^										

gnomAD genome allele frequency was obtained from v.4.0.0.

*F* female, *M* male, *Me* metopic synostosis, *S* sagittal synostosis, *BL* bilateral, *Fo* foot, *H* hand, with involved digit numbers in brackets, *ASD* atrial septal defect, *AVSD* atrioventricular septal defect, *DORV* double outlet right ventricle, *PDA* patent ductus arteriosus, *SVC* superior vena cava, *VSD* ventricular septal defect, *NA* not applicable.

^a^Work undergoing to confirm that in *trans.*

^b^No paternal sample available.

**Fig. 1:**
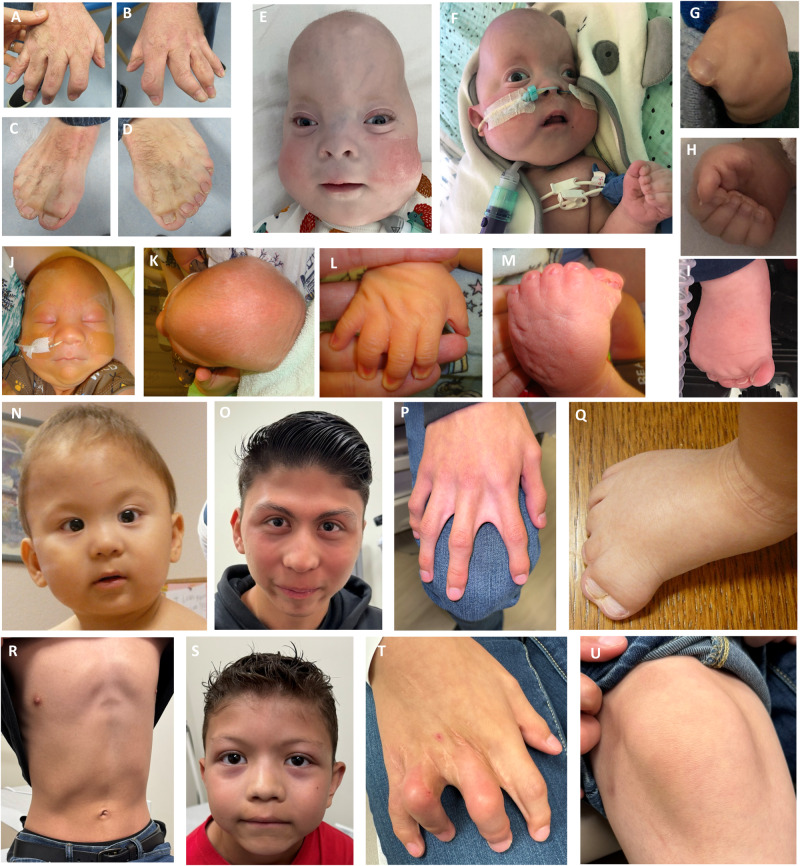
Clinical features of individuals with *MEGF8* variants. Hands (**A**, **B**; post-operative following multiple syndactyly releases) and feet (**C**, **D**) of subject 1 at the age of 37 years; facial features (**E**, **F**), hands (**G**, **H**) and feet (**I**) of subject 2.ii; facial features (**J**, **K**), hand (**L**) and foot (**M**) of subject 3; facial features (**N**, **O**), hands (**P**), feet (**Q**) and chest (**R**) of subject 6.1 at 11 months (**N**, **Q**) and 16 years (**O**, **P**, **R**); facial features (**S**), hands (**T**) and knee demonstrating broad patellae (**U**) of subject 6.ii at 10 years old.

The first patient (Subject 1) was referred to a clinical genetics department for reproductive counselling at the age of 37 years (Fig. 1A–D). He had presented during infancy with polysyndactyly and craniosynostosis, both treated surgically, and had a clinical diagnosis of CRPTS. Following negative clinical genetic testing, he was recruited to the 100,000 Genomes whole genome sequencing research project (100kGP). Initial analysis of the whole genome sequencing data did not reveal a diagnosis because the allele bearing the 3 nucleotide deletion was filtered out based on the quality threshold in operation [27], but further research investigation confirmed that he had two likely pathogenic alterations in *MEGF8*, which were shown to have been inherited biallelically. Brief details of this case were previously included in an analysis of patients with craniosynostosis in the 100kGP [27].

Family 2 comprises a foetus (Subject 2.i) terminated at 25 weeks’ gestation owing to multiple anomalies detected on ultrasound scanning, and a liveborn sibling (Subject 2.ii) (Fig. 1E-I). Post-mortem examination of the foetus documented dysmorphic facies, talipes equinovarus, hand syndactyly and abnormalities of left-right patterning. Her brother (Subject 2.ii) had antenatally-detected craniosynostosis. Post-natal findings included syndactyly, bowel malrotation with polysplenia, sagittal craniosynostosis, facial dysmorphism, and complex congenital heart disease; he required ventilation via a tracheostomy for pharyngomalacia and vocal cord paralysis. Aged two years old he has global developmental delay but is acquiring new skills. He is non-verbal but has good understanding and can communicate non-verbally, can sit and stand with support, but is not yet walking independently. Biallelically inherited *MEGF8* variants were identified in a postnatal trio exome analysis of Subject 2.ii, and the same variants were subsequently identified in a stored sample from 2.i.

Subject 3 presented antenatally with increased nuchal translucency and oedema at 20 + 3 weeks’ gestation. Post-natal findings included metopic craniosynostosis, dysmorphic facies, polysyndactyly and talipes equinovarus (Fig. 1J–M). Reinterpretation of a trio foetal exome taking account of the post-natal findings revealed two *MEGF8* variants, one maternally inherited and the other arising as a de novo variant. At the age of 20 months, he has evidence of mild speech delay and cannot walk independently, although he can walk with support and fine motor skills are unaffected.

Subject 4 presented with metopic craniosynostosis, cutaneous syndactyly of her hands and pre-axial polydactyly of her feet, together with a patent ductus arteriosus (PDA) and atrial septal defect (ASD). Aged 24 months she has delayed speech and gross motor skills. Targeted next generation sequencing with 300 gene capture and analysis of 50 genes associated with craniosynostosis identified two variants in *MEGF8*, consistent with the characteristic phenotype of Carpenter syndrome. One variant was maternally inherited, but no paternal sample was available.

Subject 5 is the 13th pregnancy of an unrelated couple with four living children, six early spontaneous miscarriages and two pregnancy losses at 20 and 23 weeks’ gestation owing to umbilical cord abnormalities. Antenatal ultrasound at 17 weeks of pregnancy demonstrated suspected craniosynostosis, short long bones, renal asymmetry and a horizontalized heart with four chambers. The couple requested a termination of pregnancy, which was performed at 20 weeks’ gestation. Post-mortem examination demonstrated dysmorphic facies, craniosynostosis of all sutures, polysyndactyly, left atrial isomerism and abdominal situs inversus, absent uterus and streak ovaries. Trio genome sequencing demonstrated biallelic alterations in *MEGF8*.

Family 6 comprises two male siblings now aged 16 (subject 6.i) and 10 (subject 6.ii) years (Fig. 1J–U). Both shared features including facial dysmorphism, cutaneous syndactyly, bilateral cryptorchidism, mild pectus deformity and developmental delay or learning difficulties. Subject 6.i additionally had metopic and sagittal synostosis and bilateral preaxial polydactyly of the feet, whilst subject 6.ii had a ventricular septal defect. Exome sequencing demonstrated biallelic variants in *MEGF8*.

### Genetic variants

**Fig. 2:**
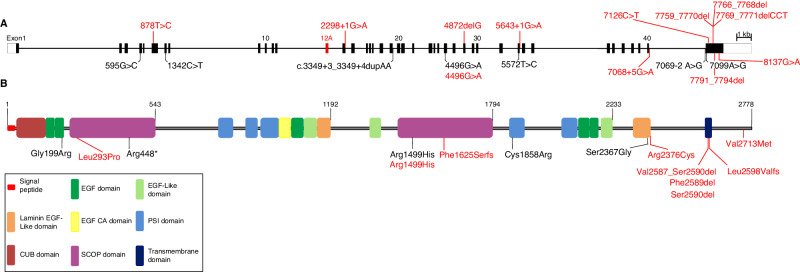
Structure of *MEGF8* and CRPT2-associated gene alterations reported in this study (red) and previously published (black). **A** Diagram of the 41 exons of *MEGF8*. The 2778 amino acid reference sequence (RefSeq accession NM_001410.3, Ensembl ENST00000334370.8) omits exon 12A, shown in red. Genetic variants are shown, previously described are shown in black, those identified in this work are shown in red. **B** Domain organisation of MEGF8 protein with previously described variants shown in black and those identified in this work shown in red.

All individuals harboured two different heterozygous variants in *MEGF8* (Table 1, Fig. 2). In four families, inheritance was demonstrated to be biallelic based upon analysis of both parents; this inheritance pattern is likely, but not formally proven in Subject 4, for whom only the maternal sample was available. Unusually for a recessive disorder, in Subject 3 one of the variants arose de novo, so the allelic relationship could not be deduced based on parental inheritance.

The twelve variants described here (Table 1, Fig. 2) comprise four missense, three in-frame deletions and five likely truncating variants (two affecting canonical splice donor sequences, one at position +5 of a splice donor sequence, and two frameshifts). Only one of these variants, (p.(Arg1499His)), has previously been reported in the literature independently of the patients in this study, being present in a homozygous state in a patient with *MEGF8*-associated CRPT2 [18]. All of the variants are either absent, or present at an extremely low frequency in the heterozygous state in the gnomAD v4.0.0 database [28]. Whilst we did not perform functional assays on any variants, the pattern of variants observed, in combination with the very distinct clinical features of CRPTS and failure to identify other plausible pathogenic variants, suggest that the phenotypes described are likely to be caused by the associated combinations of *MEGF8* variants. Regarding the three novel missense variants ((p.(Leu293Pro), p.(Arg2376Cys) and p.(Val2713Met)), all are located at extremely conserved amino acids (Supplementary Fig. 1), including invertebrates, have CADD scores >25 and are considered likely pathogenic by AlphaMissense (all three scoring 0.96 out of a maximum of 1) [29]. At position 293, vertebrate and invertebrate organisms all have a leucine or the chemically similar isoleucine. By alignment with human KEAP1, this variant locates within a β–strand of the second of six blades of a predicted Kelch domain [30] where substitution to proline is expected to be highly disruptive. The arginine at position 2376 is predicted to lie in a laminin EGF-like domain and is highly conserved across evolutionary time. Similarly, the valine at position 2713 lies within a predicted intracellular region that is very highly conserved (Supplementary Fig. 1), but does not correspond to an identified domain. It is predicted with moderate confidence by AlphaFold [31] to reside in a region of β–strand (Uniprot accession Q05BT0), but the consequence of substitution to methionine is unclear. Interestingly the three in-frame deletions, although each is distinct, all affect the same stretch of four conserved amino acids located in the single predicted helical transmembrane region (Fig. 2). No genotype-phenotype correlations are apparent. No individual harboured two loss-of-function alleles.

### Craniofacial features

Six of eight individuals had craniosynostosis, the remaining two individuals without craniosynostosis comprising a foetus terminated at 25 weeks’ gestation and a ten year old. These six cases demonstrated a spectrum of phenotypic severity, from Subject 5 who had craniosynostosis affecting all sutures detected prenatally at 17 weeks’ gestation, to Subjects 2.ii, 3 and 4 who had single suture fusion, of either the metopic or sagittal suture (Table 1). Considering all patients with *MEGF8*-associated CRPT2 to date, six out of fifteen cases have had involvement of a single midline suture, comprising five with metopic and one with sagittal craniosynostosis, and only four individuals had multiple suture craniosynostosis. In contrast, 32 out of 39 reported cases of *RAB23* CRPTS had synostosis of more than one suture and involvement of both coronal or all sutures is common [3, 4, 9–15] (Supplementary Table 2). A relatively consistent pattern of dysmorphic facial features was apparent in the series, including micrognathia, hypertelorism (including in the presence of metopic synostosis), a broad depressed nasal bridge and small low-set posteriorly rotated ears (Fig. 1).

### Limb abnormalities

All eight individuals had cutaneous syndactyly involving multiple digits of the hands, and in six individuals (Subjects 1, 2.ii, 3, 5, 6.i, 6.ii) the feet were also affected. Five individuals had polydactyly of the hands or feet and three had talipes equinovarus (Table 1, Fig. 2). Brachydactyly with missing or uncalcified middle phalanges was also present (Table 1, Supplementary Table 1). Cutaneous syndactyly is a universal feature in all cases of *MEGF8*-associated CRPT2 to date (15/15 individuals, Supplementary Table 2) and is also near-universal in *RAB23*-associated CRPT1 (38/39 individuals), with polydactyly, abnormalities of the middle phalanges and talipes also being common in both forms (Supplementary Table 2).

### Left-right patterning abnormalities

Three of eight individuals demonstrated a laterality defect, comprising both siblings from family 2, and Subject 5. In Family 2, foetus 2.i demonstrated left atrial isomerism, abdominal situs inversus, bi-lobed lungs bilaterally, midline liver and polysplenia and her surviving brother (2.ii) demonstrated complex congenital heart disease, polysplenia and bowel malrotation; whilst Subject 5 demonstrated heterotaxy with polysplenia. None of the eight individuals had dextrocardia and five did not have a left-right patterning abnormality. Considering all reported cases of *MEGF8*- and *RAB23*-associated CRPTS, laterality defects were significantly more common in CRPT2 (7/15 cases) than in CRPT1 (4/39 reported cases, Fisher’s exact test, *P* = 0.006, Supplementary Table 2).

### Prenatal features

Six of eight individuals in this series (Subjects 2.i, 2.ii, 3, 5, 6.i, and 6.ii) presented prenatally, with features of abnormal tissue fluid accumulation combined with other congenital abnormalities. These included increased nuchal translucency, polyhydramnios and congenital heart disease (Family 2); prenatal non-immune hydrops (Subject 3), increased nuchal translucency/cystic hygroma with craniosynostosis and renal asymmetry (Subject 5) and in family 6, ventriculomegaly (subject 6.i) or enlarged cisterna magna with congenital heart disease (subject 6.ii). Two pregnancies (Subjects 2.i and 5) were terminated owing to the severity of the foetal anomalies. Subject 3 had amniocentesis and prenatal exome testing, but the diagnosis was not made until the genetic data were re-examined in light of the post-natal findings. The parents of Subject 5 had six early spontaneous miscarriages and two pregnancy losses at 20 and 23 weeks’ gestation possibly owing to umbilical cord abnormalities. It is uncertain whether these foetal losses were similarly affected by CRPTS, but there was additionally a maternal history of a pituitary adenoma about which no further information is available.

### Additional clinical features

All five males in this series had cryptorchidism (Subjects 1 (unilateral), 2.ii, 3, 6.i and 6.ii, Table 1). Combined with previously reported males with *MEGF8*-associated CRPT2, all males with *MEGF8-*associated CRPTS have demonstrated cryptorchidism, which is also common but not universally reported in *RAB23*-associated CRPT1 (*MEGF8*: 9/9 males*; RAB23* 14/23 males; Fisher’s exact *P* = 0.035). Developmental delay is common, but of variable severity, ranging in this series from Subjects 2, 3 and 4 who have speech and gross motor delay, to Subject 1 who attended mainstream school with some childhood speech and language therapy and is university educated. This spectrum of severity is also reflected in the previously reported cases of both CRPT1 and CRPT2, with no clear differences depending upon causal gene (Supplementary Table 2). Congenital heart disease is present in 6/8 cases reported here, 4/7 previously reported cases of *MEGF8*-associated CRPTS and 10/39 previously reported cases of *RAB23*-associated CRPTS (Supplementary Table 2). Subject 2.ii has required long-term ventilation via a tracheostomy for respiratory difficulties, pharyngomalacia and vocal cord paralysis. Some individuals demonstrated additional skeletal features including two individuals with short long bones and three with pectus deformities or chest asymmetry. In family 6 broad patellae, camptodactyly and delayed dentition were additionally noted features and subject 6.i had a stable s-shaped scoliosis of the thoracic spine (Supplementary Table 1).

## Discussion

We describe eight individuals from six different families, each of whom harbours two different variants in the *MEGF8* gene, more than doubling the number of reported families from 5 to 11 and increasing the total number of affected individuals reported with *MEGF8*-associated CRPT2 to fifteen.

As noted above, no patient reported to date with CRPT2 has been biallelic for two loss-of function *MEGF8* alleles. Mice completely lacking *Megf8* are embryonic lethal and null mutations of the *Drosophila* homologue of *MEGF8* die as larvae [20, 21, 32], suggesting some MEGF8 function is needed for viability. Most likely, the missense and in-frame deletion alleles allow some residual function, providing rescue from early lethality. A notable feature of *MEGF8*-associated CRPT2 is the wide range of clinical severity, from prenatal presentation with multiple foetal anomalies (Subjects 2i and 5) to graduate level education (Subject 1, who is the only individual compound heterozygous for missense and in-frame deletion variants). Variable residual function associated with diverse alleles and allele combinations identified in *MEGF8*-associated CRPT2 may explain the spectrum of phenotypes seen. Also of note is that in 7 of the 11 independent families documented to date, one of the two *MEGF8* alleles has been predicted to cause complete loss of function (3 canonical splice site, 2 nonsense, and 2 frameshift substitutions). Given the rarity of *MEGF8*-related CRPT2 in the context of a very large protein target (2778 amino acids), we speculate that there may be a relatively narrow window of residual MEGF8 activity that is embryopathic, so that many homozygous or compound heterozygous combinations of missense alleles exceed a threshold of activity and are without pathogenic effect. Of interest, the gnomADv4.0.0 database lists 68 different amino acid substitutions, occurring at widely scattered locations throughout the protein, for which at least one homozygote exists. Hence we urge that great caution should be exerted in the pathogenic interpretation of biallelic *MEGF8* variants, in the absence of the characteristic clinical features described here and in previous publications [18, 23]

The CRPT2 patients reported here and previously emphasise that the core features of CRPTS are similar whether caused by *RAB23* or *MEGF8* alterations [3, 4, 9–15, 18, 23]. In particular craniosynostosis with complete cutaneous syndactyly of multiple digits is a near universal presentation, with polydactyly, cryptorchidism, talipes equinovarus, congenital heart defects and variable developmental delay being common features in both CRPT1 and CRPT2. However, left-right patterning abnormalities are a more common finding in CRPT2 [18], being present in just under half of cases to date, but only around one tenth of *RAB23*-associated CRPT1 [3, 4, 9–15]. Where laterality defects have been demonstrated in CRPT1, these are generally milder than in CRPT2 comprising only one reported individual with true abdominal situs inversus, two individuals with accessory spleens but no other evidence of heterotaxy and one individual with a double outlet right ventricle [4, 10, 12]. The clinical findings in CRPT2 mirror the heterotaxy seen in mice with *Megf8* alterations, and *megf8* knockdown in zebrafish, which was attributed to abnormal propagation of asymmetric *Nodal* expression in the lateral plate mesoderm [19]. However, 8/15 individuals with *MEGF8*-associated CRPTS do not have any evidence of a laterality defect, indicating reduced penetrance of this phenotypic feature. Even considering mouse models lacking *Megf8*, left-right patterning abnormalities were variable, with only around a half having dextrocardia or transposition of the great arteries [21], suggesting that additional unidentified factors interact with the *MEGF8* genotype to determine final left-right patterning in *MEGF8*-associated CRPTS.

Craniosynostosis is a nearly universal feature in cases of both *RAB23*- and *MEGF8*-associated CRPTS. However, the review presented here suggests that craniosynostosis in CRPT2 is often less severe. Whereas multi-suture synostosis is common in the reported *RAB23* cases, six out of the fifteen *MEGF8* cases have had documented fusion of only a single midline suture (metopic or sagittal) [18, 23], most commonly isolated metopic craniosynostosis. Nevertheless, there is clearly a spectrum of severity as one individual with CRPT2 had synostosis of all sutures and presented prenatally (case 5).

Dysmorphic facial features (hypertelorism, epicanthic folds, wide flat nasal bridge, high palate, low set posteriorly rotated ears) are shared by both types of CRPTS. These dysmorphic features appear a characteristic part of the syndrome, rather simply than a secondary effect of craniosynostosis, illustrated by the paradoxical finding of metopic synostosis with hypertelorism rather than the expected hypotelorism in cases of *MEGF8* CRPTS reported here and elsewhere [1, 18].

In conclusion, we report a further eight cases of CRPTS caused by biallelic variants in *MEGF8*, strongly supporting that CRPT2 is a distinct clinical entity. These cases confirm that *MEGF8*-associated CRPT2 shares many core features of *RAB23*-associated CRPT1, but differences include a significantly higher frequency of left-right patterning abnormalities, and lower frequency of multi-suture craniosynostosis in the *MEGF8*-associated condition, refining the phenotypic spectrum of this rare condition.

### Supplementary information

Supplemental material (final version)

Supplementary_table_1

Supplementary_table_2

## Data Availability

All data are available in the manuscript and Supplementary Material.
