# Supplementary material for: The phenotype of MEGF8-related Carpenter syndrome (CRPT2) is refined through the identification of eight new patients
Source: Eur J Hum Genet. 2024 May 17;32(7):864–70. doi: 10.1038/s41431-024-01624-9 (PMC11220001; doi:10.1038/s41431-024-01624-9)
Supplement: Supplementary file 1 — Supplemental material (final version) [file 41431_2024_1624_MOESM1_ESM.docx]

**The phenotype of MEGF8-related Carpenter syndrome (CRPT2) is refined through the identification of eight new patients** by Laura M. Watts *et al.*

1. Supplementary case descriptions (pages 1-8)
2. Supplementary Figure 1- Analysis of evolutionary conservation of Leu293Pro, Arg2376Cys and Val2713Met missense alterations in *MEGF8* (page 9)
3. Supplementary references (page 10)

**Supplementary Case Descriptions**

**Subject 1**

A 37-year-old man with a previous clinical diagnosis of Carpenter syndrome was referred for clinical genetics review prior to starting a family. He was the first child of unrelated parents of north European ancestry. There was no family history of skeletal abnormalities. He was born by emergency caesarean section weighing 3.85 kg (+0.98 SD). At birth he was noted to have fusion of the digits of his hands and feet, and polydactyly of his feet. At the age of 1 month he underwent a surgical procedure for craniosynostosis and during childhood had multiple osteotomies of his hands to separate his digits. Due to the time elapsed since his surgery, no documentation is available regarding which cranial sutures were fused. At the age of 11 years an undescended left testicle was removed. He had speech therapy as a child owing to delayed speech, but there were no other concerns regarding his development. He attended mainstream schools and university, graduating with a degree in computing.

On examination, he had a tall forehead with prominence of the metopic suture. His chest shape was asymmetrical. He had scars on his hands from previous surgery, brachydactyly and his toes remained syndactylous, with scars from removal of additional toes bilaterally. Sequencing of *RAB23* and *GLI3*, multiplex ligation-dependent probe amplification (MLPA) of *GLI3*, *ROR2* and *HOXD13*, and sequencing of hotspots in *FGFR1*, *FGFR2*, *FGFR3* and *TWIST1* associated with craniosynostosis was normal. Microarray did not demonstrate any chromosomal imbalance. He was recruited to the 100,000 Genomes whole genome sequencing research project which used an HPO-driven virtual panel based approach. Initial analysis of the whole genome sequencing data did not reveal a diagnosis, but further research interrogation confirmed that he had two likely pathogenic alterations in *MEGF8* (c.[4496G>A];[7766_7768del] encoding p.[(Arg1499His)];[(Phe2589del)]) (1). These were missed in the initial 100,000 Genomes Project analysis as the heterozygous in frame deletion was filtered out based on quality settings and the second missense variant was not tiered in the absence of a second variant being identified. Parental testing confirmed biparental inheritance.

**Subjects 2i and 2ii**

The male infant (subject 2ii) is the first liveborn child of unrelated healthy parents of north European ancestry. A previous female pregnancy (subject 2i) had been terminated at 25 weeks’ gestation owing to multiple fetal anomalies detected on ultrasound scan. Post-mortem examination of the fetus documented a dysmorphic facies (wide-set eyes, small low-set ears, small nose, flat nasal bridge, small jaw), nuchal oedema, short chest with widely spaced nipples, shortened lower limbs with talipes equinovarus, hand syndactyly, left atrial isomerism, atrio-ventricular septal defect, bilateral superior vena cavae, bi-lobed lungs bilaterally and gut malrotation (stomach on right, caecum and appendix on left of the abdomen) with polysplenia. No chromosomal imbalance was found on single nucleotide polymorphism (SNP)-array.

The male child was born at 37+2 weeks by elective caesarean section owing to polyhydramnios, weighing 3.5 kg (+0.31 SD). The pregnancy was complicated by maternal gestational diabetes treated with metformin, and polyhydramnios, ventricular septal defect (VSD), increased nuchal translucency and craniosynostosis were all detected antenatally. An antenatal SNP-array was normal. He required resuscitation after birth due to low oxygen saturations and was intubated, ventilated and admitted to the neonatal intensive care unit.

Bowel malrotation was diagnosed on the 3^rd^ day of life following abdominal distension and biliary aspirate from the nasogastric tube, treated by Ladd’s procedure and inversion appendicectomy. Post-operative recovery was complicated by wound dehiscence and infection, chylous ascites requiring drainage and intestinal obstruction necessitating further laparotomy, adhesiolysis, unkinking of the duodenum and resection of Meckel’s diverticulum. At two months of age a third abdominal surgical procedure was required for an abdominal abscess and further episode of intestinal obstruction, treated by drainage of the abscess and further adhesiolysis. This surgery was complicated by cardiovascular instability requiring inotropic support and due to his unstable condition, a high loop jejunostomy was brought out.

Following intubation at birth, continued respiratory support was needed and he was unable to wean onto non-invasive ventilation. At the age of three months a tracheostomy was performed after airway examination demonstrated circumferential pharyngomalacia and left vocal cord paralysis. He remains on long term ventilation via the tracheostomy. Post-natally, an echocardiogram confirmed complex congenital heart disease (double outlet right ventricle (DORV), VSD, atrial septal defect (ASD), and persistent ductus arteriosus (PDA)), with preserved biventricular systolic function. The PDA was ligated at nearly two months old with banding of the pulmonary artery. Aged 20 months old he had surgical repair of the DORV, closure of the ASD, pulmonary artery de-banding and main pulmonary artery reconstruction.

Additional features included scaphocephaly, later confirmed by 3-dimensional computed tomography scanning as related to sagittal synostosis, dysmorphic facial features (short nose with anteverted nares, micrognathia, short neck with redundant skin, small low-set ears), brachydactyly, cutaneous syndactyly of all digits of the hands and feet (without polydactyly), polysplenia and bilateral undescended testes. The cutaneous syndactyly was treated by release of the web spaces in several procedures. Surgical treatment of the craniosynostosis is planned for age 15-24 months. On most recent review aged 17 months, he had global developmental delay but was acquiring new skills, could bottom shuffle and use a standing frame. He had exotropia, with reduced visual acuity of 6/12 in both eyes.

Rapid gene agnostic, inheritance based trio whole exome sequencing was performed, which revealed that the child was a compound heterozygote for two likely pathogenic variants in *MEGF8* (c.[878T>C];[2298+1G>A] encoding p.[(Leu293Pro)];[(?)]), Table 1). Subsequent testing of the sister’s stored sample confirmed that she carried the same compound heterozygous variants in *MEGF8*.

**Subject 3**

This patient, the first child of unrelated parents, presented with multiple fetal abnormalities on ultrasound scan at 20+3 weeks’ gestation. Further ultrasound at a specialist fetal medicine centre at 21+2 weeks’ gestation demonstrated a pericardial effusion, increased nuchal fold and significant facial and lower limb oedema. Middle cerebral artery Doppler readings were normal, providing no evidence for fetal anaemia. Amniocentesis was performed at 21+2 weeks with no genetic abnormality detected on rapid aneuploidy testing, microarray or trio whole exome sequencing.

The male baby was born at 38+4 weeks’ gestation after spontaneous onset of labour. Birth weight was 3.375 kg (+0.06 SD) and length was 52 cm (+1.12 SD). Postnatally, he was reviewed by the clinical genetics team on the neonatal intensive care unit. Findings were of suspected metopic suture craniosynostosis with a prominent metopic ridge and bitemporal narrowing; dysmorphic facial features (short upslanting palpebral fissures, bilateral epicanthic folds, prominent nasal root with broad ridge, micrognathia, high arched narrow palate, low set and posteriorly rotated ears), and abnormalities of the hands and feet. The hands showed cutaneous syndactyly, broad thumbs and brachydactyly due to rudimentary/missing middle phalanges demonstrated on plain radiographs. The feet demonstrated bilateral symmetrical pre-axial polydactyly, brachydactyly, cutaneous syndactyly and bilateral talipes equinovarus. Other features included a closing PDA with minimal aortic root dilatation, small renal cortical cysts, left-sided hydronephrosis, and bilateral cryptorchidism. His electroencephalogram (EEG) was abnormal at birth with dysrhythmic background which resolved, and cranial imaging showed tiny cysts in the caudothalamic groove. He did not have seizures.

The patient was treated with and front-orbital advancement and anterior vault cranial remodelling at seven months old, in addition to bilateral orchidopexy. The PDA closed during the first year of life, with minimal aortic root dilatation persisting at 20 months. The renal cysts and hydronephrosis resolved by 20 months, leaving left renal hypodysplasia with normal renal function. At 20 months old, he has evidence of mild speech delay and cannot walk independently, although he can walk with support and fine motor skills are unaffected.

The prenatal exome was reinterpreted in the context of the postnatal clinical phenotype. Two variants were identified in *MEGF8*. One is a *de novo* splice site alteration (c.5643+1G>A) predicted to cause loss of the splice donor site. The other (c.8137G>A, p.Val2713Met) is maternally inherited. It is located in a highly conserved region of *MEGF8* (Supplementary figure 1). Work is ongoing to confirm that these variants are in *trans*.

**Subject 4**

This girl was born weighing 3.95 kg (+1.47 SD) to non-consanguineous parents. She was noted to have trigonocephaly due to metopic craniosynostosis. She additionally had cutaneous syndactyly of all digits of her hands and bilateral pre-axial polydactyly of her feet. Cardiac features were of a PDA and ASD. She failed her newborn hearing screen. At 24 months old her height is 82 cm (-1.37 SD) and weight is 12.9 kg (+0.93 SD). She has delayed speech and gross motor skills.

Targeted next generation sequencing with 300 gene capture and analysis of 50 genes associated with craniosynostosis identified two variants in *MEGF8.* One (c.7769_7771del, p.Ser2590del) is maternally inherited. No paternal sample was available at the time of testing, but work is ongoing to confirm paternal origin of the other variant (c.4872delG, p.Phe1625Serfs*38).

**Subject 5**

This was the 13th pregnancy of an unrelated couple, who had four living children, six early spontaneous miscarriages and two pregnancy losses at 20 and 23 weeks’ gestation due to umbilical cord abnormalities. The third child had a cleft palate and scoliosis. Mother had a pituitary adenoma diagnosed in 2005 (not removed, further information unavailable), previous appendicitis and cholecystectomy. The father has no medical, surgical, or notable familial history.

Ultrasound performed at 12 weeks of the pregnancy showed that the craniocaudal length was 63 mm (consistent with a 12 week 5 day pregnancy) and the nuchal translucency was increased at 6.8 mm (>99^th^ centile for the craniocaudal length). Two days later repeat ultrasound confirmed a cystic hygroma measuring 9 mm together with cranial, thoracic and abdominal subcutaneous oedema. Further ultrasound examination at a specialist prenatal centre at 17 weeks of pregnancy demonstrated an abnormal skull shape with suspected craniosynostosis, enlargement of the pericerebral spaces, deviation of the heart axis to the left, length of the long bones below the 1st percentile and renal asymmetry. Echocardiography showed a horizontalized heart with four chambers. An amniocentesis was performed for a chromosomal microarray, which did not demonstrate any chromosomal imbalance. The couple requested a termination of pregnancy, which was performed at 20 weeks.

The fetopathological examination confirmed multiple congenital malformations in a female fetus. Facial dysmorphism consisted of upslanted palpebral fissures, hypertelorism, highly arched eyebrows, anteverted naries, long philtrum, and low-set posteriorly rotated ears. There was craniosynostosis of all sutures together with polysyndactyly of all four extremities and thumb brachymetacarpy. The fetus demonstrated abdominal situs inversus with left atrial isomerism, supernumerary spleen, short pancreas and uterine agenesis with banded ovaries. The long bones were short with notably large and stubby femurs and there was left renal hypoplasia. There was additionally an extra-cranial dermo-epidermal lesion of the scalp with skin hypertrophy and capillary hyperplasia. Trio genome sequencing revealed compound heterozygous variants in *MEGF8* (c.[7759_7770del];[7791_7794del] encoding p.[(Val2587_Ser2590del)];[(p.Leu2598Valfs*24)]).

**Subjects 6.i and 6.ii**

Family 6 comprises two affected male siblings currently aged 16 (subject 6.i) and 10 (subject 6.ii) years. The older sibling was born at term to 20-year-old unrelated Hispanic parents who had previously experienced five first trimester losses. Evaluation for these losses had revealed a maternal balanced 13;14 translocation. The mother declined prenatal diagnosis during her pregnancy with 6.i for choroid plexus cysts and mild ventriculomegaly. His birth weight was 4.37 kg (+1.64 SD). He had an unusual skull shape and craniosynostosis was documented involving the sagittal and metopic sutures. He had bilateral preaxial polydactyly and syndactyly of his feet and mild cutaneous finger syndactyly. He underwent neurosurgical release of the cranial sutures and plastic surgery for removal of the preaxial great toes in infancy. He also had bilateral cryptorchidism and inguinal hernias repaired in infancy. Development was mildly delayed and he has had attention deficit hyperactivity disorder (ADHD). Educationally he has been in normal classes with some support. He had delayed dental eruption, severe dental caries and at age 16 years still retains several primary teeth.

His physical examination at age 16 years reveals tall stature (height 186 cm (+2 SD)), normal weight (73 kg +0.93 SD), and a head circumference of 54.8cm (-0.20 SD) with a slightly short forehead, aberrant frontal hair patterning, sparse lateral eyebrows, long philtrum, significant micrognathia, and dental malocclusion. He has prominent trapezius muscles, an asymmetric chest with displacement of the left nipple, a mild carinatum deformity and slightly widened umbilicus. He has broad patellae and a stable s-shaped thoracic scoliosis. Hands reveal spatulate thumbs and unrepaired syndactyly with mildly restricted joint motion (camptodactyly) at the proximal interphalangeal joints. His feet show repair of the syndactyly and scars from removal of the preaxial digits.

The younger sibling 6ii, currently 10 years old, was born at 39 weeks after a pregnancy complicated by ultrasound suspicion of congenital heart disease and an enlarged cisterna magna. Prenatal diagnosis was declined. His birth weight was 4.76 kg (+2.4 SD). A large VSD was documented and he underwent open repair at two months old. He did not have craniosynostosis. He had surgery for pyloric stenosis at 3 weeks of age and for cryptorchidism before the age of two years. He had significant 2-5 finger syndactyly and 5^th^ finger clinodactyly repaired in infancy and mild toe syndactylies (unrepaired). He has had delayed loss of primary teeth with currently only two permanent teeth. He had mildly delayed development with walking at 18 months and single words at age 2 years. At age 10 he is in normal classes but functioning 1-2 years below his chronological age and he requires additional educational support. At age 10 years his height is 146 cm (+1.1 SD), weight is 39.5 kg (+0.83 SD) and head circumference 51.6 cm (-0.94 SD). He has mild hypertelorism, sparse eyebrows laterally, long philtrum and mild micrognathia. He has multiple missing primary teeth. He has prominent trapezius muscles. Significant finger contractures (camptodactyly) followed release of his syndactylies. Patellae are broad bilaterally. He has a midline sternotomy scar and a somewhat long and broad umbilicus.

Both 6i and 6ii were found to be carriers of the maternal balanced 13;14 translocation. Following recruitment to the Genetic Basis of Craniofacial Malformation study, exome sequencing was performed on a DNA sample from Subject 6.ii, employing the same general methods as reported by Miller et al.(2), with the NimbleGen SeqCap EZ Exome Library v2.0 used for exome capture. Following alignment with Bowtie2 and variant calling with Samtools v1.1, two single nucleotide variants with allele frequency <0.001 were identified in *MEGF8*. Further investigation of the family showed that Subjects 6i and 6ii were both compound heterozygous for these *MEGF8* variants (c.[7126C>T];[7068+5G>A] encoding p.[(Arg2376Cys)];[(?)]); the variants were shown to have been paternally and maternally inherited, respectively. According to SpliceAI(3) and Pangolin(4) the 7068+5G>A variant, which locates at a selectively constrained nucleotide(5), is predicted to disrupt splicing at the donor splice site with scores 0.23 and 0.45, respectively.

**
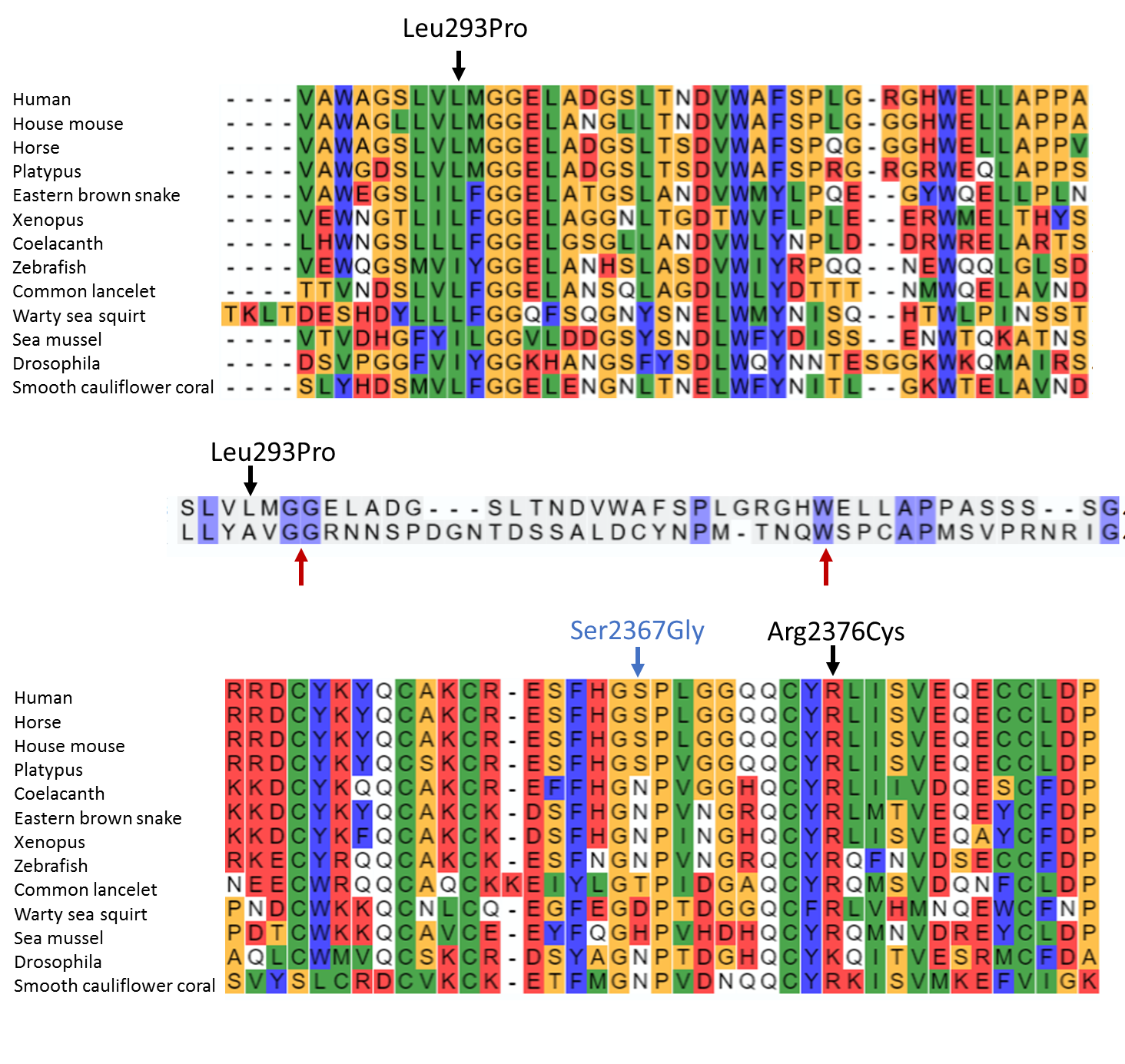
**
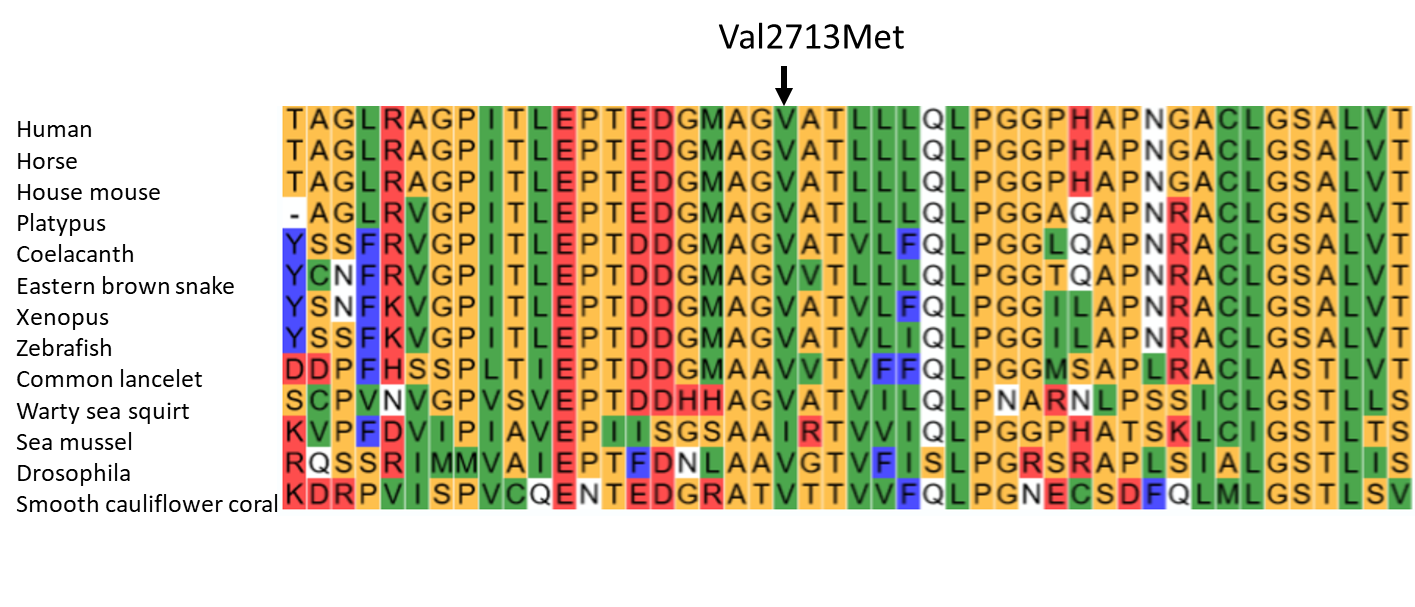


**Supplementary figure 1: Analysis of evolutionary conservation of Leu293Pro, Arg2376Cys and Val2713Met missense alterations in *MEGF8*.**

Protein sequences for organisms from human to invertebrates were retrieved from UniProt and aligned using the Clustal Omega algorithm. Positions of three human missense variants are labelled, demonstrating deep evolutionary conservation at these sites. At position 293, which locates within a Kelch domain, all displayed organisms have a leucine or the chemically similar isoleucine. Alignment of the Kelch2 domains of MEGF8 and human KEAP1, a canonical Kelch domain containing protein, is shown. The conserved adjacent glycines and later tryptophan of the Kelch domain are marked by red arrows. At position 2376 all displayed organisms have an arginine, other than Drosophila which has a lysine, which is also a positively charged basic amino acid. The position of the pathogenic missense variant Ser2367Gly, previously reported by Twigg et al.(6) and located nearby, is also shown in blue. At position 2713, all displayed organisms have a valine other than the sea mussel which has isoleucine, again a conservative substitution. The deep evolutionary conservation at all three sites, including invertebrates, supports the pathogenicity of the corresponding missense substitutions.

**Supplementary references**

1. Hyder Z, Calpena E, Pei Y, Tooze RS, Brittain H, Twigg SRF, et al. Evaluating the performance of a clinical genome sequencing program for diagnosis of rare genetic disease, seen through the lens of craniosynostosis. Genet Med. 2021;23:2360–8.

2. Miller KA, Twigg SRF, McGowan SJ, Phipps JM, Fenwick AL, Johnson D, et al. Diagnostic value of exome and whole genome sequencing in craniosynostosis. J Med Genet. 2017;54:260–8.

3. Jaganathan K, Kyriazopoulou Panagiotopoulou S, McRae JF, Darbandi SF, Knowles D, Li YI, et al. Predicting Splicing from Primary Sequence with Deep Learning. Cell. 2019;176:535-548.

4. Zeng T, Li YI. Predicting RNA splicing from DNA sequence using Pangolin. Genome Biol. 2022;23:103.

5. Lord J, Gallone G, Short PJ, McRae JF, Ironfield H, Wynn, EH, et al. Pathogenicity and selective constraint on variation near splice sites. Genome Res. 2019;29:159-170.

6. Twigg SRF, Lloyd D, Jenkins D, Elçioglu NE, Cooper CDO, Al-Sannaa N, et al. Mutations in multidomain protein MEGF8 identify a Carpenter syndrome subtype associated with defective lateralization. Am J Hum Genet. 2012;91:897–905.
